# Probabilistic forecasts of trachoma transmission at the district level: A statistical model comparison

**DOI:** 10.1016/j.epidem.2017.01.007

**Published:** 2017-03

**Authors:** Amy Pinsent, Fengchen Liu, Michael Deiner, Paul Emerson, Ana Bhaktiari, Travis C. Porco, Thomas Lietman, Manoj Gambhir

**Affiliations:** a Department of Public Health and Preventative Medicine, Monash University, Melbourne, Australia; b F.I. Proctor Foundation, University of California San Francisco, San Francisco, CA, USA; c International Trachoma Initiative, Atlanta, GA, USA; d School of Public Health, Emory University, Atlanta, GA, USA; e Department of Ophthalmology, University of California San Francisco, San Francisco, CA, USA; f Department of Epidemiology and Biostatistics, University of California San Francisco, San Francisco, CA, USA; g Global Health Sciences, University of California San Francisco, San Francisco, CA, USA

**Keywords:** Trachoma, Elimination, Forecasting, Model comparison

## Abstract

The World Health Organization and its partners are aiming to eliminate trachoma as a public health problem by 2020. In this study, we compare forecasts of TF prevalence in 2011 for 7 different statistical and mechanistic models across 9 de-identified trachoma endemic districts, representing 4 unique trachoma endemic countries. We forecast TF prevalence between 1–6 years ahead in time and compare the 7 different models to the observed 2011 data using a log-likelihood score. An SIS model, including a district-specific random effect for the district-specific transmission coefficient, had the highest log-likelihood score across all 9 districts and was therefore the best performing model. While overall the deterministic transmission model was the least well performing model, although it did comparably well to the other models for 8 of 9 districts. We perform a statistically rigorous comparison of the forecasting ability of a range of mathematical and statistical models across multiple endemic districts between 1 and 6 years ahead of the last collected TF prevalence data point in 2011, assessing results against surveillance data. This study is a step towards making statements about likelihood and time to elimination with regard to the WHO GET2020 goals.

## 1 Introduction

Trachoma remains the world’s leading infectious cause of blindness (Anon, 2012, Mariotti et al., 2009), and it is currently estimated that 200 million individuals are living at risk of blindness from trachoma (World Health Organization, 2016). WHO and its partners are aiming to eliminate trachoma as a public health concern by 2020. To help achieve this, WHO endorses the SAFE strategy. This four pronged approach includes: Surgery for trichiasis, Antibiotics, particularly mass treatment with azithromycin of all residents of endemic districts, Facial cleanliness, and Environmental improvement (Taylor et al., 2012).

Elimination of trachoma as a public health problem has a definition with two component goals. The first is to reduce the prevalence of trachomatous inflammation- follicular (TF) in children 1–9 years old to <5% at the district level by 2020. The second is to reduce the prevalence of trachomatous trichiasis cases to <1/1000 at the district level. In this article we focus on the achievement of the first goal outlined.

Mathematical and statistical modelling continues to be used for a wide range of infectious diseases, including trachoma (Gebre et al., 2012, Lietman et al., 2011, Liu et al., 2013, Gambhir et al., 2009, Blake et al., 2009, Pinsent et al., 2016a). Studies are conducted to help understand and quantify epidemiological outcomes following clinical trials, and to assess the impact of different treatment interventions. In trachoma, detailed randomised control trial (RCT) data have been analysed and modelled with statistical and dynamic models (Lietman et al., 2011, Liu et al., 2013, Liu et al., 2015a, Lietman et al., 1999, Liu et al., 2015b) to assess and predict the outcomes of given interventions. Such models can also be used to estimate the resource requirements to achieve certain goals, such as elimination or the achievement of specific disease prevalence/incidence thresholds (Hollingsworth et al., 2015, Stolk et al., 2015a, Wouters et al., 2014, Stolk et al., 2015b, Turner et al., 2016). While producing highly informative and accurate forecasts for an infectious disease is challenging, it is desirable from a public health perspective. This is because they enable high priority regions to be identified and help to develop an understanding of the resources required in different areas in order to achieve the proposed targets.

Mathematical and statistical models are used to make predictions about the future prevalence and incidence of infectious diseases. However, predictions from individual models are not commonly tested robustly against other potential forecasts, nor are they regularly validated against independent data. Nevertheless, robust statistical model comparison of outcomes from different models is essential, in order to understand the limitations and strengths of different mathematical and statistical modelling approaches. Most commonly in trachoma, the Susceptible, Infected, Susceptible (SIS) model structure has been used (Lietman et al., 2011, Liu et al., 2013, Gambhir et al., 2009, Liu et al., 2015a, Lietman et al., 1999, Liu et al., 2015b, Ray et al., 2007, Ray et al., 2009), though variants of this structure have also been proposed (Pinsent et al., 2016a, Liu et al., 2015b, Shattock et al., 2015). Liu et al. (Liu et al., 2015a) conducted a statistical model comparison assessing a variety of statistical and mechanistic models. Fitting each model to PCR data, the authors found that statistical regression models and SIS mechanistic models performed significantly better than expert opinion. This suggests that the use of mathematical and statistical modelling may be useful in projecting trachoma prevalence (Liu et al., 2015a).

In this study we compare the probabilistic forecasts of TF prevalence generated by statistical (without trachoma-specific assumptions), mechanistic (SIS and partially acquired immunity) and mixed (SIS plus a random effect) models to TF prevalence estimates determined empirically in field based surveys, for 9 de-identified trachoma endemic districts. Probabilistic prevalence forecasts are scored as the log-likelihood of the observed 2011 data for each district given the model, allowing us to ascertain the strengths and weaknesses of different modelling approaches to make forecasts of TF prevalence.

## 2 Methods

### 2.1 Data

We used de-identified district level TF prevalence data collected and shared by the International Trachoma Initiative (ITI). These data contained district level TF prevalence at 1 or more time points, collected between the years 1995–2010. Data after 2010 were available; however, all forecasters were masked to it. Within this dataset, information regarding the number of rounds of MDA provided and the years in which they were administered were also provided for each district. Three of the 4 models we evaluated used country-level parameters or random effects. These parameters were estimated from the ITI data collected between 2001 and 2010. These were data from 43 countries which included 1037 unique districts, of which: 953 had a single survey, 82 had 2 surveys, 1 had 3 surveys, and 1 had 4 surveys.

To estimate the district-level random effects for the SIS and statistical regression models all district-level data in the ITI database collected between 1995 and 2010 was used. This included data on 1107 unique districts, of which: 918 had a single survey, 171 had 2 surveys, 17 had 3 surveys, and 1 had 4 surveys; 189 unique districts with at least 2 surveys were used to estimate the district-level random effects.

**Fig. 1:**
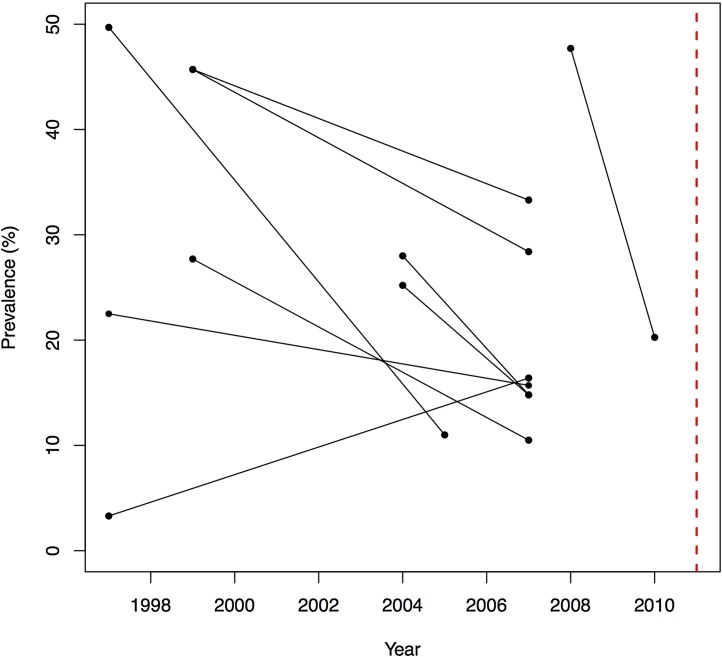
District level TF prevalence in each of the 9 districts between 1997 and 2010. Every set of two data points and one line indicates the prevalence data for that district. The red dashed like indicates the forecasted year of 2011.

The district-level transmission coefficients for the deterministic transmission model used a total of 9 districts for which TF prevalence data were present for at least two time points between 1995 and 2010, and for which a TF prevalence data point was also available for 2011. As the data were de-identified by country and district it was not possible to know the population size of the district at each sampling time point. For each of the 9 districts we then forecast the distribution of TF prevalence in 2011. Trends in prevalence over time for each district are presented in Fig. 1, where each line represents a separate district. Across 8/9 districts we observed a gradual decline in TF prevalence following initiation of antibiotic treatment. We note, that for all districts follow-up was infrequent and hence we have used a much smaller subset of the data to fit the models. Data on TF prevalence and years they are reported for is presented in Table 1.

### 2.2 Mathematical models

#### 2.2.1 Model 1: deterministic transmission model

The first type of model evaluated was an age-structured deterministic ordinary differential equation (ODE) transmission model. We used a model structure that has been statistically chosen (Pinsent and Gambhir, 2017) as the most appropriate and parsimonious when fitting to cross-sectional PCR and TF prevalence data (West et al., 2005). We consider individuals as susceptible to infection (*S*), exposed and incubating (*E*), infected and infectious with detectable TF (*AI*) and those who remain diseased but no longer infectious to others (*D*), individuals in the *D* state are susceptible to re-infection with a reduced probability. Those who were re-infected then returned to the *AI* state. We hereafter refer to this model as Model 1. A schematic diagram of this model structure is presented in Fig. S1.

The model followed a previously-detailed ‘ladder of infection’ structure (Gambhir et al., 2009, Pinsent et al., 2016a, Pinsent et al., 2016c), which accounted for the development of immunity to infection through successive infections (immunity here was represented as a reduction in an individual's infectivity and a faster rate of recovery from infection and disease with each successive infection). We assumed that both the infectivity and that the duration of infection and disease decreased exponentially with each successive infection. Further detail on the model structure and parameters used are provided in the supplementary information and Table S1.

For each of the 9 districts we performed a Markov Chain Monte Carlo (MCMC) search of the 2D parameter space to estimate and explore the uncertainty in both estimated parameters (MDA coverage and *β*). We then sampled 100 pairs of values from the posterior distribution to explore the inherent uncertainty in the 2011 forecast generated. Additionally, we also took 100 sets of samples from the posterior distributions when our model was fitted to cross-sectional PCR and TF data (West et al., 2005) which estimated the minimum rate of recovery from an individual's first infection and disease episode. Forecasts of the 2011 prevalence were thus generated incorporating uncertainty in the minimum rate of recovery from infection and disease as well as variation in the estimated value of *β* and coverage. This was done to ensure that we explored the uncertainty in the natural history parameters and how these uncertainties may impact the 2011 forecast. All calculations were performed in the R 3.2.1 (R Core Team, 2015), the package deSolve was used to solve the differential equation model (Soetaert et al., 2010).

#### 2.2.2 Model 2: mixed mechanistic and statistical model

We also assessed a mixed-effect SIS model, of which there were three different types, hereafter referred to as Models 2.1, 2.2 and 2.3 respectively. 2.1) a model with country-district-level random effect given all-district data, 2.2) a model with country-district-level random effect given 9-district data, and 2.3) a model with country-district-level random effect and treatment given 9-district data.

2.1) To fit the SIS model with country-level and district-level random effects given all-district data: for each district, we used the first available year’s data (between 1995 and 2010) from that district as the initial prevalence, and predicted the prevalence in each year (until 2010), and then obtained the likelihood of the data in the rest of the available years for that district; the total likelihood was obtained from all districts’ likelihoods. The model’s parameters were estimated using MCMC (details on the calculation of the likelihood are provided in the supplementary material). To include the country-level and district-level random effects, we assumed that the prior of the transmission coefficient was the same for every country; the posterior of transmission coefficient within a country was obtained by weighting with the likelihood of data from that country. The prior of the transmission coefficient in each district of a country was the same and from that country's posterior; the posterior of the transmission coefficient in a district of that country was obtained by weighting with the likelihood of data from that district. With the posterior of the transmission coefficient in each district, we forecasted TF prevalence in each of the 9 districts in 2011.

2.2) The SIS model with country-level and district-level random effects given 9-district data, was similar to the first model; we used the 9-district data instead of all-district data to fit the model. For Models 2.1 and 2.2 a parameter for treatment was not explicitly modelled and, instead, the effects of treatment were assumed to be captured by the transmission coefficient parameter. Through this parameter we capture variation in prevalence through time, thus indirectly accounting for antibiotic treatment.

2.3) The SIS model with two-level random effects: a country level random effect and a district level random effect, and treatment (given 9-district data) was similar to the second model. We used an efficacy parameter to represent treatment. Prediction by SIS model was based on the initial prevalence with the treatment efficacy included. To model the implementation of treatment, we assumed that, for a given year, a district received treatment if TF prevalence data was reported in the same year for that district, otherwise we assumed no treatment was given to that district in that year as the prevalence point was only a follow-up survey.

We modelled treatment for a district in a given year according to $p_{i}=\overset{N}{\underset{i^{\prime }=i}{\sum }}p_{i^{\prime }}^{\left(pre\right)}\left(\begin{array}{c} i^{\prime }\\ i \end{array}\right){\left(1-ef\right)}^{i}ef^{i^{\prime }-i}$, where *i'* is the number of infected people with probability $p_{i^{\prime }}^{\left(pre\right)}$ before treatment, *i* is the number of infected people after treatment, *ef* is the product of treatment efficacy and coverage (fixed value assumed), and *N* is the population size of the district. To simplify the computation N = 100 in each district was assumed. Parameters for the SIS mixed-effects model are presented in Table S2. All calculations were performed in the R programming language (R Core Team, 2015).

#### 2.2.3 Model 3: district-agnostic statistical forecasts

A single forecast distribution was used for all 9 districts. This distribution was determined in two ways. In the first, the 2-parameter, zero-inflated exponential distribution that had the highest likelihood of the observed district-level prevalence data for each year from 2001 to 2010 was found. Here we estimated the scale parameter and the proportion of districts with a prevalence of zero. A linear regression on these parameters for each year was then used to extrapolate the 2 parameters for the 2011 zero-inflated exponential. The single forecast for all 9 districts was thus made from a 2-parameter model. In the second, the 3-parameter, zero-inflated gamma distribution that had the highest likelihood of the observed district-level prevalence data for each year from 2001 to 2010 was found. Here we estimated the shape and scale parameters in addition to the proportion of communities with a prevalence of zero. A linear regression on these parameters for each year was used to extrapolate parameters for the 2011 zero-inflated gamma distribution. The single forecast for all 9 districts was thus made from a 3-parameter model. All calculations were performed in Mathematica 11.2 (Wolfram Research, Champaign, Illinois). These models are hereafter referred to as Model 3.1 and 3.2.

#### 2.2.4 Model 4: district-specific statistical forecasts

A mixed effects regression model was used on log-transformed data from before 2011. Covariates included district-level clinical activity (TF or TF/TI), survey type (prevalence survey, school survey, or trachoma rapid assessment), and year, with normally distributed random intercepts at the country and region level. This model did not incorporate any understanding of the natural history of infection. District-specific forecasts were then made from this 5-parameter model. All calculations were performed in the R programming language (R Core Team, 2015) using the package lmer (Bates et al., 2015). Uncertainty analysis was conducted using the Metropolis algorithm. This model is hereafter referred to as Model 4.

#### 2.2.5 Scoring

To evaluate the performance of each model’s probabilistic forecast, in comparison to the data, we calculated the log-likelihood (LL), also known as the Ignorance Score (Good, 1952, Carney and Cunningham, 2006). Our pre-specified score was the LL of the observed 2011 TF prevalence data at the district level. This score was calculated as the logarithm of the likelihood of the observed data given the model.

We calculated the score for each individual district level forecast with two different approaches. The forecasts (calculated as% prevalence of TF) and the observed data were binned into 101 units for the first scoring approach, with the bins delineated by the [0,0.5), [0.5,1.5), [1.5,2.5), …, [99.5, 100]. For the second scoring approach the data and forecasts were binned as: [0.5,1.5), [1.5,2.5), …, [99.5, 100]. The rationale for doing both was that a high number of districts worldwide are known to be zero or near zero, and are therefore not being surveyed. If we had universal surveying in our data, the [0,0.5) bin would be very high, and conditioning our forecast distribution on being greater than 0 may be more relevant. As 3 of the 9 districts evaluated had a very low prevalence in 2011 but 6 did not, we present the scores from both calculations.

Calculation of the LL for each model forecast for each district was as follows. For each bin for a single district we had a data (TF prevalence) value for that bin (0 if the data was not in that particular bin, and 1 if the data was in that bin) and we also had each models forecasted prevalence distribution within that bin for that district. Therefore, to obtain the LL for each bin for a single model, for a single district, we calculated the following LL = data*LN(forecasted), where data was 0 for all bins, except the one bin that the data falls into, which was 1. Forecasted values were between 0 and 1 (and the total of all the values from all the bins for a district forecast equals 1.000000). Then for the individual district for each model forecast, we summed the LL values across all bins, to obtain the LL Score for that district. All calculations were performed using Microsoft Excel.

We calculate *p*-values using the Wilcoxon signed-rank test for the comparison between different models using the first scoring method only, as this approach includes the score from all 9 districts and the second approach only includes 6 districts.

## 3 Results

**Fig. 2:**
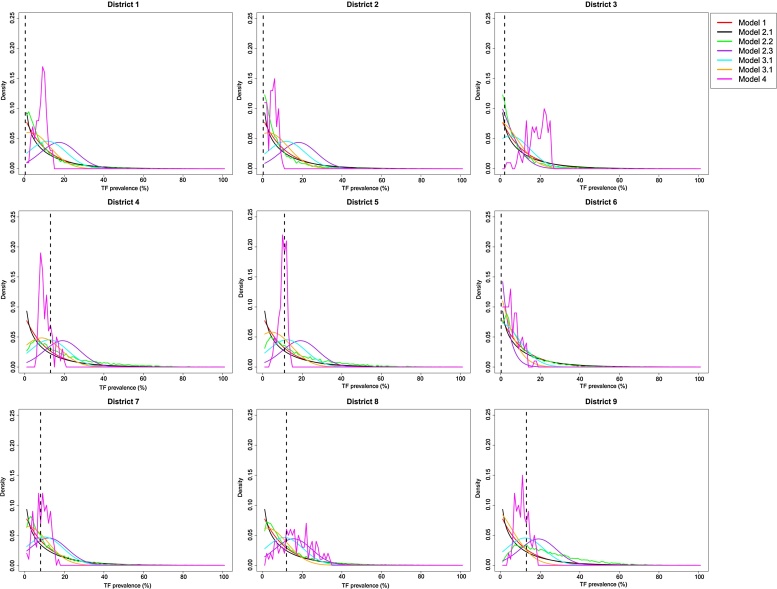
Forecast distributions of TF prevalence in 2011 for each of the 9 districts evaluated and for each of the 7 models analysed. Results from model forecasts are shown by a solid line and the true data for 2011 for each district is shown with a black dashed line. The colour of each line represents a different model as indicated in the legend.

The best performing model across all 9 districts (as assessed by the highest calculated LL score) was the SIS model with country-district-level random effects and treatment, given 9-district data (Model 2.3, Table 2). All model forecasts across the 9 districts are presented in Fig. 2. This result was consistent across both scoring methods (Tables 2 and S3). It was followed very closely by Model 3, where the gamma distribution (Model 3.2) was slightly better performing than the exponential model. However, the results from Model 3 were statistically indistinguishable from one another in terms of their LL scores −27.08 and −26.96 for each model respectively (*p* = 0.77, Table 2). The next best performing model was Model 4, the mixed-effects regression model. We note that for models 2–4 the overall LL scores were not significantly different from one another. Comparing Model 2.3 vs Model 2.2, *p* = 0.31, Model 2.3 vs Model 4, *p* = 0.17 and Model 2.3 vs Model 3.1, *p* = 0.06. The least well performing model (highest LL score), across all 9 districts collectively, was Model 1, with a LL score of −51.84 (Table 1), markedly worse than the other three models.

Evaluating the performance of each model at the individual district level, we saw that for Models 2–4, the value of the LL was rarely less than −3.5, suggesting a consistent performance at the individual district level (Table 1). However, these models only very rarely achieved extremely high LL values, highlighting that while they were able to predict the data well, their concentration of probability mass was usually not in the same bin as the actual data. For Model 1 there was slightly more heterogeneity in the LL scores between districts (Table 1), with the worst performance seen in district 3. In districts 2, 4–9 the LL scores were relatively low (all >-3.5, Table 1), with the LL score of >-3.9 for district 1. Indicating that for the majority of districts the performance of Model 1 was comparable to Models 2–4. However for district 3 using Model 1, the LL score was −29.93 (Table 1). This district began at a very high initial TF prevalence and declined substantially over the intervention period. As such, the dramatic declines in prevalence observed in this district could not be captured with Model 1.

Assessing the density distributions for each district for all models (Table 2, Fig. 2), Model 1 consistently produced the narrowest TF prevalence forecast (also the least smooth, due to the use of only 100 replicates). Model 1, 2 and 4 resulted in different projections across each of the different districts. While Model 3.1 and 3.2 provided the same forecast across all districts (Table 2, Fig. 2) and in general, the peak of all forecast distributions from these models was towards zero (Table 2, Fig. 2). Models 2–4 forecast a wider distribution of TF prevalence values, in comparison to the more narrow forecasts of Model 1. Assessing the findings in Table 3, for districts 1–3, Models 2.3–4 all had a high probability mass (>0.25) between the forecasted TF prevalence of: 0–4.9, 5.0–<9.9% and 10.0–< 29.9% (Table 3). Suggesting that all TF prevalence outcomes were almost equally likely. This wide variation in forecasted TF prevalence was consistent for these models across all districts (Table 2 and Table 3), but to a slightly lesser extent for Model 4. For districts 1–3 the data indicated no intervention was required, therefore at least 50% of the time Models 2.3-4 would have substantially over-estimated the possible intervention effort required. In comparison, for district 5, >0.70 of the probability mass of Model 1 and 2.1 fell within the range of the observed data (Table 3) and would have correctly estimated the additional level of intervention required in the district.

## 4 Discussion

In this study we have provided long-term probabilistic forecasts of TF prevalence from longitudinal surveillance data across multiple endemic districts (Liu et al., 2015b), and compared our findings across a variety of different mathematical and statistical models. Overall the SIS model with country-district-level random effects and treatment (Model 2.3) was the best performing model, although the projected uncertainty covered a large range of prevalence values. However, Model 2.3 was not significantly better than the district-specific mixed-effects regression model or the non-district-specific overall distribution (Models 3 or 4). The deterministic transmission model (Model 1) had the lowest total log-likelihood score across all 9 districts; however, it produced results for 8 out of 9 districts that were close to the better performing models. Therefore, overall forecasts that utilized district-specific information (Models 1 and 2) did not perform statistically better than the district-agnostic Model 3. The duration of our projected forecasts ranged between 1 and 6 years, allowing us to study our models’ ability to capture long-term trends in TF prevalence. For models 2–4, performance was not impacted by the duration of the forecast (Table 2 and 3). However, for Model 1 the forecast for district 3 was the worst performing, and it was this district that required the longest forecast duration. Suggesting that the long duration of the forecast may have impacted this model’s performance.

Our forecasts have been made at the district level, and therefore pertain directly to assessing the goal of reducing TF prevalence in children 1–9 to <5% in all endemic districts by 2020. Across all 9 districts we identified sustained declines in TF prevalence following MDA. Indeed, for 4 out of 9 districts TF was below 5% in 2011 and, for 5 out of 9 (which had higher starting prevalence of TF), TF had still declined. Therefore this study forecasting district level TF prevalence (although at times with large uncertainty) is the first step towards making statements about likelihood and time to elimination with regard to the WHO GET2020 goals.

For the majority of districts Model 1 performed comparably well to Models 2–4; however, it struggled to predict low prevalence’s, particularly when the initial reported TF prevalence was very high. It is challenging to fit deterministic models to low incidence settings. However, it would be possible to include a non-linear transmission term to help overcome this issue as it would slow the dynamics down near a breakpoint and also allow for elimination (Lietman et al., 2011). Alternatively, MDA is only one component of the SAFE strategy, therefore it could be that transmission reduction measures through F and E have also helped to reduce prevalence in high transmission settings (Pinsent et al., 2016b). However, currently no data are available to us on the implementation of F and E within these districts.

Models 2–4 were able to perform consistently well across all districts, as evaluated by the high LL score, even though the uncertainty associated with each forecast resulted in a much wider expected distribution of TF prevalence values. For Model 2 the absolute range of the forecast was ∼45%, and slightly higher for Models 3 and 4 (Fig. 2). Most commonly, when forecasts are made, how close the point estimate of the data is to the highest probability mass of the forecast is the most important outcome. However, when forecasting the effect/impact of an intervention or allocating resource optimally, the uncertainty associated with the outcome can also be important. If there is large uncertainty surrounding the possible impact of a given intervention, from a programmatic perspective we may apprehensive about investing in the intervention. Indeed, we observed that with some forecasts we would have substantially overestimated the effort required to reach TF <5% in some districts where TF was already <5%, particularly with Models 1, 2.1 and 2.2 (Table 2). However, for a number of districts which showed TF <5% in 2011 Models 3.1, 3.2 and 2.3 underestimated the additional level of intervention that would be required. This suggests that such models may not be as helpful in districts where prevalence of TF remains >5%. These models had a wide forecast of TF prevalence, suggesting that TF <5%–29.5% were almost equally likely.

The scoring metric used here was the LL of the forecast distribution (also known as the ignorance score). The LL of the observed data fulfils the requirements of a proper score, including that if the forecast coincided with the observations themselves, the optimal score would be achieved (Bröcker and Smith, 2007). However, the LL score is a negatively orientated score; which means that it weights results that have only a low forecast probability heavily. Additionally, this approach evaluates density estimates solely based on the probability density at the true observation point, and does not take the calibration of the forecast into account (Soetaert et al., 2010). The LL score is just one of several proper scores that could be used. An alternative scoring approach may be a quadratic score which would not penalize poor single forecasts as severely, in comparison to the approach used here.

Across all models there is room for improvement to enhance the performance of future forecasts. Our models represent a simplification of a complex and dynamic biological process. Therefore, it is possible that some components of the system are missing within the existing modelling frameworks, and these could be incorporated in further work. For example, transmission may reduce over time as a consequence of environmental or socio-economic improvements; however, this has not been considered in any of the models evaluated here, and could be incorporated with a time varying transmission parameter, instead of the fixed value used here. Equally, for the district-agnostic forecasts, alternative distributions could be tested with this data, such as the beta distribution. Limited data exist to validate the assumed levels of treatment coverage in the model, therefore it is also possible that these estimates could be improved. Evidence has suggested that antibiotic treatment with azithromycin can have an anti-inflammatory effect, and thus may reduce TF. This has not may been explicitly modelled and inclusion of this may help to improve Model 1′s forecasts. Additionally, while we have used the best available data on global trachoma prevalence, the data could be considered sparse in terms of the frequency of follow-up as we are only forecasting from two previous prevalence points. Therefore more frequent follow-up surveillance data prior to the forecasted time point may help us understand the dynamics of disease better. Nevertheless, while it is tempting to assume that additional data would improve our forecasts, it should be noted that apparently similar neighbouring communities often have markedly different prevalence, and that the prevalence in the same community over time can vary considerably. Thus, additional data may only complicate forecasting further.

It is important to note that the data used here were collected through a number of heterogeneous survey approaches. In addition, there is also variation in the TF grader training methodologies. While we acknowledge this limitation of the data it was not possible to quantify or account for biases in that data that may be present due to this heterogeneity. Such variation may have resulted in inconsistencies in the wider dataset that could not be accounted for. Nevertheless, in the future, as a result of greater international standardization in both of these things, we will have more reliable data on which we can base predictive models.

We have demonstrated a statistical model comparison scheme that allows a range of mathematical and statistical models to be evaluated and compared. As a next step we would look to forecast multiple time points for which frequent follow-up data are available, in order to provide further validation for our models. However, it may be that larger datasets with more districts and frequent longitudinal follow-up will be required if we wish to be able to statistically distinguish the performance of different modelling approaches. Nevertheless, our results provide hope that different mathematical models can be used to forecast trachoma successfully in the future.

## Figures and Tables

**Table 1:** Data on TF prevalence and the years the data was collected from the 9 anonymised districts evaluated in the study.

District	Sample date year 1	Sample prevalence year 1 (%)	Sample date year 2	Sample prevalence year 2 (%)	Sample date year 3	Sample prevalence year 3 (%)
1	1997	22.7	2007	15.7	2011	0.1
2	1997	3.3	2007	16.4	2011	1.2
3	1997	49.7	2005	11	2011	3.7
4	1999	45.7	2007	33.3	2011	12.8
5	1999	45.7	2007	28.4	2011	10.5
6	1999	27.7	2010	10.5	2011	4.5
7	2004	25.5	2007	13.5	2011	8.3
8	2004	28.0	2007	14.8	2011	11.9
9	2007	40.7	2010	20.2	2011	12.5

**Table 2:** The log-likelihood (LL) score for each model and each district evaluated for both scoring methods applied is provided. We also present the total LL score for each model across the total 9 districts analysed. We present within each row for each model the values derived from the first scoring method (with 101 bins).

Model type	District 1 LL score*	District 2 LL score*	District 3 LL score*	District 4 LL score*	District 5 LL score*	District 6 LL score*	District 7 LL score*	District 8 LL score*	District 9 LL score*	Total LL score*
Model 1	−3.912	−2.040	−29.933	−2.659	−1.660	−3.506	−2.659	−2.813	−2.659	−51.84
Model 2.1	−4.105	−4.277	−2.397	−3.342	−3.502	−0.881	−3.181	−3.192	−3.343	−28.23
Model 2.2	−3.129	−3.175	−2.950	−3.097	−3.100	−1.911	−3.073	−3.078	−3.100	−26.61
Model 2.3	−2.115	−2.176	−2.592	−3.137	−3.132	−1.336	−2.951	−3.261	−3.268	−23.43
Model 3.1	−2.321	−2.321	−2.653	−3.634	−3.456	−2.321	−3.188	−3.545	−3.634	−27.08
Model 3.2	−2.000	−2.000	−2.627	−3.801	−3.633	−2.005	−3.364	−3.718	−3.801	−26.96
Model 4	−3.122	−2.743	−2.198	3.450	−3.320	−3.673	−2.990	−3.458	−3.348	−28.31

**Table 3:** For each model and district we present that probability mass of forecasted TF prevalence, which fell within the intervals closest to the ITI programmatic thresholds for intervention. * indicates that the true data fell within this interval.

Model type	District evaluated								
	District 1	District 2	District 3	District 4	District 5	District 6	District 7	District 8	District 9
**Model 1**									
0–4.9%	0.18 *	0.56 *	0.04 *	<0.001	<0.02	0.48	0.20	0.05	0.02
4.5–9.9%	0.54	0.43	0.05	0.51	0.37	0.35*	0.40 *	0.15	0.40
10.0–29.9%	0.28	0.01	0.91	0.48 *	0.61*	0.16	0.40	0.73 *	0.58 *
30.0–49.9%	<0.001	<0.001	<0.001	0.01	<0.001	0.01	<0.001	0.07	<0.001
50%+	<0.001	<0.001	<0.001	<0.001	<0.001	<0.001	<0.001	<0.001	<0.001

**Model 2.1**									
0–4.9%	0.07 *	0.06 *	0.50 *	0.05	0.05	0.84	0.15	0.09	0.06
4.5–9.9%	0.12	0.10	0.24	0.10	0.10	0.13 *	0.18 *	0.15	0.10
10.0–29.9%	0.71	0.72	0.26	0.72 *	0.72 *	0.03	0.60	0.68 *	0.73 *
30.0–49.9%	0.10	0.12	<0.001	0.11	0.11	<0.001	0.07	0.08	0.11
50%+	<0.001	<0.001	<0.001	<0.001	<0.001	<0.001	<0.001	<0.001	<0.001

**Model 2.2**									
0–4.9%	0.15 *	0.15 *	0.28 *	0.14	0.14	0.38	0.19	0.18	0.14
4.5–9.9%	0.20	0.20	0.26	0.20	0.20	0.28 *	0.23 *	0.22	0.20
10.0–29.9%	0.60	0.60	0.40	0.61 *	0.61 *	0.28	0.54	0.57*	0.61 *
30.0–49.9%	0.05	0.05	0.02	0.05	0.06	0.06	0.04	0.03	0.05
50%+	<0.001	<0.001	<0.001	<0.001	<0.001	<0.001	<0.001	<0.001	<0.001

**Model 2.3**									
0–4.9%	0.38 *	0.38 *	0.48 *	0.25	0.36	0.65	0.41	0.39	0.51
4.5–9.9%	0.27	0.27	0.27	0.24	0.27	0.22 *	0.27 *	0.27	0.26
10.0–29.9%	0.35	0.35	0.25	0.50 *	0.37 *	0.12	0.32	0.34 *	0.23 *
30.0–49.9%	<0.001	<0.001	<0.001	0.01	<0.001	0.01	<0.001	<0.001	<0.001
50%+	<0.001	<0.001	<0.001	<0.001	<0.001	<0.001	<0.001	<0.001	<0.001

**Model 3.1**									
0–4.9%	0.39 *	0.39 *	0.39 *	0.39	0.39	0.39	0.39	0.39	0.39
4.5–9.9%	0.22	0.22	0.22	0.22	0.22	0.22*	0.22*	0.22	0.22
10.0–29.9%	0.32	0.32	0.32	0.32*	0.32*	0.32	0.32	0.32*	0.32*
30.0–49.9%	0.07	0.07	0.07	0.07	0.07	0.07	0.07	0.07	0.07
50%+	<0.001	<0.001	<0.001	<0.001	<0.001	<0.001	<0.001	<0.001	<0.001

**Model 3.2**									
0–4.9%	0.40 *	0.40 *	0.40 *	0.40	0.40	0.40	0.40	0.40	0.40
4.5–9.9%	0.22	0.22	0.22	0.22	0.22	0.22*	0.22*	0.22	0.22
10.0–29.9%	0.32	0.32	0.32	0.32*	0.32*	0.32	0.32	0.32*	0.32*
30.0–49.9%	0.06	0.06	0.06	0.06	0.06	0.06	0.06	0.06	0.06
50%+	<0.001	<0.001	<0.001	<0.001	<0.001	<0.001	<0.001	<0.001	<0.001

**Model 4**									
0–4.9%	0.41*	0.50 *	0.50 *	0.16	0.20	0.37	0.35	0.31	0.06
4.5–9.9%	0.24	0.23	0.23	0.20	0.21	0.26 *	0.26 *	0.25	0.15
10.0–29.9%	0.28	0.22	0.22	0.43 *	0.41 *	0.31	0.33	0.36 *	0.50 *
30.0–49.9%	0.07	0.05	0.05	0.15	0.13	0.05	0.05	0.06	0.23
50%+	<0.001	<0.001	<0.001	0.06	0.05	0.01	0.01	0.02	0.07
