# Supplementary material for: Probabilistic forecasts of trachoma transmission at the district level: A statistical model comparison
Source: Epidemics. 2017 Mar;18:48–55. doi: 10.1016/j.epidem.2017.01.007 (PMC5340843; doi:10.1016/j.epidem.2017.01.007)
Supplement: Supplementary file 1 [file mmc1.docx]

Supplementary material: Probabilistic forecasts of trachoma transmission at the district level: a statistical model comparison

**Modelling methods Model 1 – mechanistic transmission model**

Following infection in the *S* state, individuals progress into the *I* class at a rate $\lambda$, this rate of removal from *S* to *I* is independent of infection history, hence an individual’s susceptibility to re-infection is independent of their previous infection. Individuals progress out of *I* at rate $\gamma$_i_ to the *AI* class where they are infectious and also present with active disease; they progress out at a rate $\sigma$_i_ and hence recover from infection. Here the rate of recovery from infection does depend on the number of previous infections. Individuals recover from *AD* at a rate $\zeta$_i_, which also depends on the number of previous infections experienced. A schematic of Model 1 is presented in Figure S1.


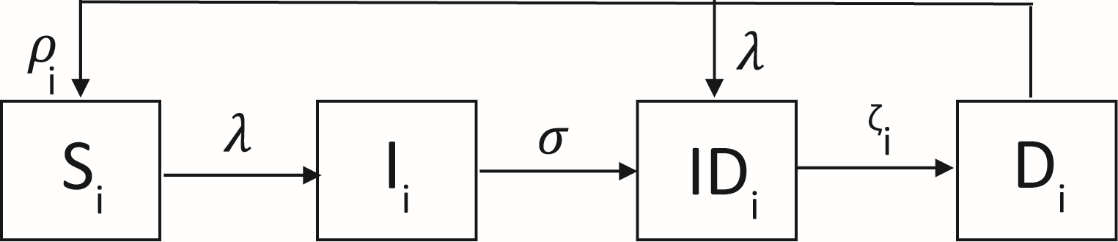


Figure S1. A schematic flowchart of model 1. Individuals can be in 1 of 4 states. Susceptible S, exposed (E) with an active infection (PCR and TF positive) (AI) or in a disease only state (TF positive only) (D). With each successive infection individuals develop immunity through reduced probability of transmission and faster rates of recovery from infection and disease. Individuals can be re-infected in the D state and return to the AI state, or they can recover naturally from disease and return to the susceptible state.

Equations for the age-structured deterministic model are as follows:

$$\begin{matrix} \frac{\partial S_{i}}{\partial t}+\frac{\partial S_{i}}{\partial a}=-\lambda_{(a)}S_{i}\left(a,t\right)-\mu_{(a)}S_{i}\left(a,t\right)+\zeta_{i-1}AD_{i-1}\left(a,t\right)+\mu_{(a)}\delta\left(i=1\right) \\ \frac{\partial I_{i}}{\partial t}+\frac{\partial I_{i}}{\partial a}=\lambda_{(a)}S_{i}\left(a,t\right)-\mu_{(a)}I_{i}\left(a,t\right)-\gamma_{i}I_{i}\left(a,t\right) \\ \frac{\partial AI_{i}}{\partial t}+\frac{\partial AI_{i}}{\partial a}=\gamma_{i}I_{i}\left(a,t\right)-\mu_{(a)}AI_{i}\left(a,t\right)-\sigma_{i}AI_{i}\left(a,t\right) \\ \frac{\partial AD_{i}}{\partial t}+\frac{\partial AD_{i}}{\partial a}=\sigma_{i}AI_{i}\left(a,t\right)-\mu_{(a)}AD_{i}\left(a,t\right)-\zeta_{i}AD_{i}\left(a,t\right) \end{matrix}$$

Where $\lambda$ is defined as:

$\rho_{j}$ is the infectivity of an individual in compartment *AI_j_*. $\gamma$ is the rate at which infected individuals $I_{i}$ progress to becoming infected and infectious, $\sigma_{i}$ is the rate at which ${IA}_{i}$ individuals recover from infection, $\zeta_{i}$ is the rate at which ${AD}_{i}$ individuals recover from active disease only. $w\left( a,a^{'} \right)$ is a mixing matrix which contains information on the rate of mixing between individuals of age group a and a’ [1].

$$w\left( a,a^{'} \right)= \varepsilon\delta_{a,a'}+(1- \epsilon)\frac{N_{a'}}{\sum_{a'} N_{a'}}$$

$\delta_{a,a'}$ is the Kronecker Delta. *N_a’_* is the number of individuals of age a’ in the population and $\varepsilon$ indicates the degree of mixing assortativity, which can range between 0 (random age mixing) and 1 (fully assortative).

**Rate of recovery**

The per individual rate of recovery from infection $\sigma_{i}$ (measured as a rate per year) from infection *i* the rate of recovery is assumed to change as an exponential function of *i* that begins at a rate of $\sigma_{1}$ (recovery from the first infection) and rises to a maximum rate $\sigma_{100}$ where no greater rate of recovery can be achieved after this point.[2]

$\sigma_{i}= {(\sigma}_{1}- \sigma_{100})\exp\left[ -\omega\left( i-1 \right) \right]+ \sigma_{100}$

We also model the per individual rate of recovery from active disease only $\zeta_{i}$ (measured as a rate per year) from infection *i* the rate of recovery is assumed to change as an exponential function of *i* that begins at a rate of $\zeta_{1}$ (recovery from the first infection) and rises to a maximum rate $\zeta_{100}$. We assume the rate of change of the recovery rate per infection $(\omega)$ is the same for recovery from infection and active disease.

$$\zeta_{i}= {(\zeta}_{1}- \zeta_{100})\exp\left[ -\varphi\left( i-1 \right) \right]+ \zeta_{100}$$

Values of these parameters are provided in Table S1.

**Infectivity**

We assumed the infectivity of an individual $(\rho_{j})$ was proportional to the log of their bacterial load $l_{i}$, which was a function of the number of previous infections experienced by each individual, a trend that is in agreement with the data from trachoma endemic communities in which the bacterial load decreases with age [5,6]. We assumed that an individual’s load decreased with an increasing number of infections experienced. We assumed a linear decline in the log of the bacterial load. This function also saturated after 100 infections had been experienced.

**Treatment of individuals**

We modelled treatment so that individuals in *I_i_* who are treated successfully return to the *D_i_* state. However, for those in *E_i_*, if they were treated successfully we assumed that they then moved into *S_i_*  state, therefore they did not develop any immunity as a result of exposure to the pathogen. We assumed a fixed efficacy of treatment and estimated coverage to estimate the transmission rate parameter beta and the level of coverage within the community

**Table S1.** Parameters and state variables used in Model 1. Numbers in brackets show the range of values encompassed in the forecasts of Model 1

| **Name** | **Definition** | **Value** | **Units** | **Source** |
| --- | --- | --- | --- | --- |
| *S_i_* | State variable |  |  |  |
| *E_i_* | State variable |  |  |  |
| *AI_i_* | State variable |  |  |  |
| *D_i_* | State variable |  |  |  |
| *c* | Coverage level of treatment | Estimated | Percentage |  |
| $\theta$ | Susceptibility to re-infection in the disease state | 0.2 | Proportion |  |
| *e* | Efficacy of antibiotic | 80% | Percentage | [3, 4] |
| $\varepsilon$ | Degree of random mixing in the population | 0.5 | Proportion | [2, 5] |
| *µ* | Birth/death rate | 1/60 | Years^-1^ |  |
| $\gamma$ | Rate at which infected individuals become infectious | 1/5 | Days^-1^ | [6] |
| $\sigma_{1}$ | Minimum rate of recovery from 1^st^ infection | 1/155  (1/121 – 1/321) | Days^-1^ | [6] |
| $\sigma_{100}$ | Maximum rate of recovery from 100^th^ infection | 1/77 | Days^-1^ | [6] |
| $\zeta_{1}$ | Minimum rate of recovery from active disease after 1^st^ infection | 1/300 (1/273 – 1/346) | Days^-1^ | [6] |
| $\zeta_{100}$ | Maximum rate of recovery from active disease after 100^th^ infection | 1/7 | Days^-1^ | [6] |
| $\rho$ | Infectivity of an individual proportional to the log of their bacterial load | Range from 0-1 | Proportion | [2] |
| $\beta$ | Transmission rate parameter | Estimated | Days^-1^ |  |
| $\omega$ | Rate of change of the recovery rate per infection | 0.45 | Infection ^-1^ | [6] |
| $\varphi$ | Rate of change of the recovery rate per disease episode | 0.30 | Infection ^-1^ | [6] |
| N_infs | Maximum number of infections before immunity saturates | 100 | Number |  |

In order to estimate the transmission rate parameter beta and coverage for each district we maximize the product of the binomial likelihood across all TF prevalence values for each district.

**Modelling methods Model 2 – SIS random effects model**

We used an SIS (susceptible-infectious-susceptible) model structure, assuming that the force of infection is proportional to the prevalence of infection in the population with proportionality constant $\beta$, and a constant per-capita recovery rate $\gamma$. The probability $p_{i}$ that there are $i$ infections in a district (population size = $N$) at time $t$ obeys the following equations:

$\frac{dp_{0}}{dt}=\gamma p_{1}$ (1)

$\frac{dp\_i}{dt}=\beta\frac{\left( i-1 \right)\left( N-i+1 \right)}{N}p_{i-1}+\gamma\left( i+1 \right)p_{i+1}-\beta\frac{i\left( N-i \right)}{N}p_{i}-\gamma ip_{i}$, for $1\leq i\leq N-1$ (2)
 $\frac{dp_{N}}{dt}=\beta\frac{N-1}{N}p_{N-1}-\gamma Np_{N}$ (3)

As shown in Equation 4, the likelihood of all available data $D$ given transmission coefficient $\beta$, $p\left( D | \beta\right)$, is the sum of the product of the weight of country-level transmission coefficient $\beta_{k}$ given $\beta$, $p\left( \beta_{k} | \beta\right)$, and the total likelihood of the data from each country $D_{j}$ given $\beta_{k}$ , $\prod_{j} p\left( D_{j} | \beta_{k},\beta\right)$, assuming that $p\left( D_{j} | \beta_{k},\beta\right)=p\left( D_{j} | \beta_{k} \right)$. The likelihood of each country, $p\left( D_{j} | \beta_{k},\beta\right)$, is the sum of the product of the weight of district-level transmission coefficient $\beta_{k}^{(i)}$ given $\beta_{k}$ and $\beta$, $p(\beta_{k}^{(i)}|\beta_{k},\beta)$, and the total likelihood of the data from each district $D_{j}^{(m)}$ given $\beta_{k}^{(i)}$, $\prod_{m} p\left( D_{j}^{(m)} | \beta_{k}^{(i)}, \beta_{k},\beta\right)$, assuming that $p\left( D_{j}^{(m)} | \beta_{k}^{(i)}, \beta_{k},\beta\right)=p\left( D_{j}^{(m)} | \beta_{k}^{(i)} \right)$, where $\beta_{k}^{(i)}$ is assumed to be in a normal distribution $\mathcal{N}\left( \beta_{k}^{(i)}; \beta_{k}, \sigma^{2} \right)$, and $\beta_{k}$ in an exponential distribution $Exp\left( \beta_{k}; 1/\beta\right)$.

$p\left( D | \beta\right)=\sum_{k} p\left( \beta_{k} | \beta\right)\prod_{j} p\left( D_{j} | \beta_{k},\beta\right)=\sum_{k} p\left( \beta_{k} | \beta\right)\prod_{j} \sum_{i} p(\beta_{k}^{(i)}|\beta_{k},\beta)\prod_{m} p\left( D_{j}^{(m)} | \beta_{k}^{(i)}, \beta_{k},\beta\right)$ (4)

Table S2: Estimated parameters

| Parameters* | SIS model 1 | | | SIS model 2 | | | SIS model 3 | | |
| --- | --- | --- | --- | --- | --- | --- | --- | --- | --- |
|  | Mean | 95% CI lower | 95% CI upper | mean | 95% CI lower | 95% CI upper | mean | 95% CI lower | 95% CI upper |
| SD of $log\beta$ | 0.93 | 0.53 | 1.00 | 0.81 | 0.52 | 0.99 | 0.78 | 0.50 | 0.99 |
| $\gamma$ | 0.49 | 0.48 | 0.50 | 0.47 | 0.42 | 0.50 | 0.47 | 0.42 | 0.50 |
| $e$ | - | - | - | - | - | - | 0.65 | 0.51 | 0.79 |
| $\beta$ | 0.04 | 0.00 | 0.08 | 0.11 | 0.00 | 0.35 | 0.10 | 0.00 | 0.35 |

***** $\beta$: transmission coefficient, $\gamma$: recovery rate (month^-1^), $e$: product of treatment efficacy and coverage.

Table S3: We present within each row for each model the values derived from the second scoring method (with 100 bins). – indicates that a score was not given for that district because in 2011 prevalence was 0%.

| **Model type** | **District 1 LL score*** | **District 2 LL score*** | **District 3 LL score*** | **District 4 LL score*** | **District 5 LL score*** | **District 6 LL score*** | **District 7 LL score*** | **District 8 LL score*** | **District 9 LL score*** | **Total LL score*** |
| --- | --- | --- | --- | --- | --- | --- | --- | --- | --- | --- |
| Model 1 | - | - | -29.913 | -2.659 | -1.660 | - | -2.649 | -2.813 | -2.659 | -42.36 |
| Model 2.1 | - | - | -2.146 | -3.331 | -3.491 | - | -3.140 | -3.169 | -3.332 | -18.61 |
| Model 2.2 | - | - | -2.851 | -3.056 | -3.060 | - | -3.018 | -3.030 | -3.052 | -18.07 |
| Model 2.3 | - | - | -2.420 | -3.069 | -3.018 | - | -2.815 | -3.133 | -3.482 | -17.94 |
| Model 3.1 | - | - | -2.550 | -3.531 | -3.353 | - | -3.085 | -3.442 | -3.531 | -19.49 |
| Model 3.2 | - | - | -2.483 | -3.656 | -3.488 | - | -3.219 | -3.578 | -3.656 | -20.08 |
| Model 4 | - | - | -2.120 | -3.441 | -3.311 | - | -2.966 | -3.440 | -3.347 | -18.63 |

**References**

1. Anderson R, May R: **Infectious diseases of humans: dynamics and control**: Oxford University Press.

2. Gambhir M, Basanez MG, Burton MJ, Solomon AW, Bailey RL, Holland MJ, Blake IM, Donnelly CA, Jabr I, Mabey DC *et al*: **The development of an age-structured model for trachoma transmission dynamics, pathogenesis and control**. *PLoS Negl Trop Dis* 2009, **3**(6):e462.

3. Liu F, Porco TC, Mkocha HA, Munoz B, Ray KJ, Bailey RL, Lietman TM, West SK: **The efficacy of oral azithromycin in clearing ocular chlamydia: mathematical modeling from a community-randomized trachoma trial**. *Epidemics* 2014, **6**:10-17.

4. Keenan JD, Ayele B, Gebre T, Zerihun M, Zhou Z, House JI, Gaynor BD, Porco TC, Emerson PM, Lietman TM: **Childhood Mortality in a Cohort Treated With Mass Azithromycin for Trachoma**. *Clinical Infectious Diseases* 2011, **52**(7):883-888.

5. Gambhir M, Basanez MG, Turner F, Kumaresan J, Grassly NC: **Trachoma: transmission, infection, and control**. *The Lancet infectious diseases* 2007, **7**(6):420-427.

6. Grassly NC, Ward ME, Ferris S, Mabey DC, Bailey RL: **The Natural History of Trachoma Infection and Disease in a Gambian Cohort with Frequent Follow-Up**. *Plos Neglected Tropical Diseases* 2008, **2**(12).
