# Supplementary material for: Probabilistic forecasts of trachoma transmission at the district level: A statistical model comparison
Source: Epidemics. 2017 Mar;18:48–55. doi: 10.1016/j.epidem.2017.01.007 (PMC5340843; doi:10.1016/j.epidem.2017.01.007)
Supplement: Supplementary file 4 [file mmc4.docx]

***Mathematica 11.0* (Wolfram Research, Champaign)**

**Fitting zero-inflated gamma distribution**

list4=Table[Cases[list3,x_/;x[[2]]<=year && x[[2]]>year-d],{year,Table[y,{y,2010,2000,-d}] } ];

list20=Table[

testList=Transpose[list4[[j]] ][[1]]/100.;

{α,β,γ}/.(NMaximize[{,α>0,β>0,γ>0,γ<1},{α,β,γ}])[[2]],{j,1,Length[list4]}];

{αα=Fit[Table[{i,list20b[[i,1]]},{i,1,Length[list20b]}],{1,x},x]/.x->0,

ββ=Fit[Table[{i,list20b[[i,1]]list20b[[i,2]]},{i,1,Length[list20b]}],{1,x},x]/.x->0,

γγ=Fit[Table[{i,list20b[[i,3]]},{i,1,Length[list20b]}],{1,x},x]/.x->0}
